# Socioeconomic disparities in the burden of hypertension among Indonesian adults - a multilevel analysis

**DOI:** 10.1080/16549716.2022.2129131

**Published:** 2022-10-11

**Authors:** Yusuf Ari Mashuri, Nawi Ng, Ailiana Santosa

**Affiliations:** a Faculty of Medicine, Universitas Sebelas Maret, Surakarta, Indonesia; b School of Public Health and Community Medicine, Institute of Medicine, Sahlgrenska Academy, University of Gothenburg, Gothenburg, Sweden; c Department of Epidemiology and Global Health, Faculty of Medicine, Umeå University, Umeå, Sweden

**Keywords:** Inequality, hypertension, wealth index, concentration index, concentration curve

## Abstract

**Background:**

Hypertension remains a problem of public health across various socioeconomic groups, despite its high prevalence. However, few studies account for geographical variation in examining socioeconomic inequalities and hypertension in Indonesia.

**Objective:**

To investigate the burden of hypertension in Indonesia based on prevalence, awareness, treatment, and control of hypertension among adults; and assess whether or not the burdens vary according to geographical variation and socioeconomic status

**Methods:**

In Wave 5 of the Indonesian Family Life Survey in 2015, 32,034 individuals aged 15 and over participated in the study. Concentration Curves (CC) and Concentration Indexes (CI) were used to analyse socioeconomic inequality. We used multilevel logistic regression to assess biological, geographical variation, and socioeconomic factors associated with the burden of hypertension, adjusting for potential covariates.

**Results:**

The prevalence of hypertension in Indonesia was 26.1%, and only 26.9% of those with hypertension were aware of their condition. Approximately 22.5% of hypertensive patients received treatment, but only 28.2% had controlled blood pressure and reached the therapeutic goal. Low socioeconomic groups were more prone to hypertension (CI = −0.047 in urban and CI = −0.075 in rural). In contrast, awareness, treatment, and control of hypertension were more concentrated in higher socioeconomic groups.

**Conclusions:**

The high prevalence of hypertension, low awareness of the condition, poor compliance with treatment, and poor control of the condition, as well as the existing socioeconomic inequality, make this a significant determinant of public health issue in Indonesia. There is a need for effective programs for the prevention of hypertension and better management of hypertensive patients.

## Introduction

Hypertension prevalence has risen dramatically in recent decades. The prevalence of hypertension doubled between 1975 and 2015 [1]. One in four people suffers from hypertension, one of the leading causes of non-communicable diseases (NCD) mortality. By 2025, this number is expected to reach 1.5 billion [1]. As Southeast Asia countries undergo rapid modernization and change in lifestyles, they are likely to experience epidemiological changes related to hypertension in the next few decades, especially as their populations approach those of developed countries, which places noncommunicable diseases like hypertension at the forefront.

In Southeast Asia, one-third of adults are currently diagnosed with hypertension, and over one million hypertension-related deaths occur annually [2]. A recent systematic review reported a 33.8% prevalence of hypertension in Southeast Asian urban populations [2]. Furthermore, modifiable factors (physical activity, smoking, dyslipidemia, alcohol consumption, body mass index, waist circumference) and nonmodifiable factors (male, ethnic group, a family history of hypertension) are all common risk factors for hypertension [2–6].

Of the Southeast Asian countries, Indonesia, the largest and most populated country in the region, has the highest prevalence of hypertension (42.7% of men and 39.2% of women). In addition, awareness, treatment, and control of hypertension remain low [3,4,7]. A study conducted in Indonesia examined socio-demographic inequalities in the diagnosis and management of hypertension in Indonesian adults using Indonesia Family Life Survey data in 2007 and found that 67% of adults with hypertension were unaware that they had hypertension, less than 30% take antihypertensive drugs, and just 25% have controlled blood pressure [8]. As a result, these worrying numbers potentially contribute to the global burden and the DALY of hypertension.

Earlier studies on ecological data in Indonesia found lower socioeconomic status was associated with higher hypertension [9,10]. Residents of urban areas are more aware of their condition than those in rural areas. Males living in urban areas in Indonesia have a lower life expectancy than those in rural areas, which is related to BMI and systolic blood pressure [9]. Nevertheless, findings on the socioeconomic status impact on hypertension in Indonesia are limited [5,11,12], particularly regarding how socioeconomic inequality affects hypertension treatment and management in Indonesia [13,14]. Moreover, only a few studies on socioeconomic inequality utilise the concentration index and concentration curve We, therefore, aim to fill the evidence gap by investigating the burden of hypertension in Indonesia based on prevalence, awareness, treatment, and control of hypertension among adults; and assess whether or not the burdens vary according to socioeconomic status.

## Methods

### Study design and population

**Figure 1.**
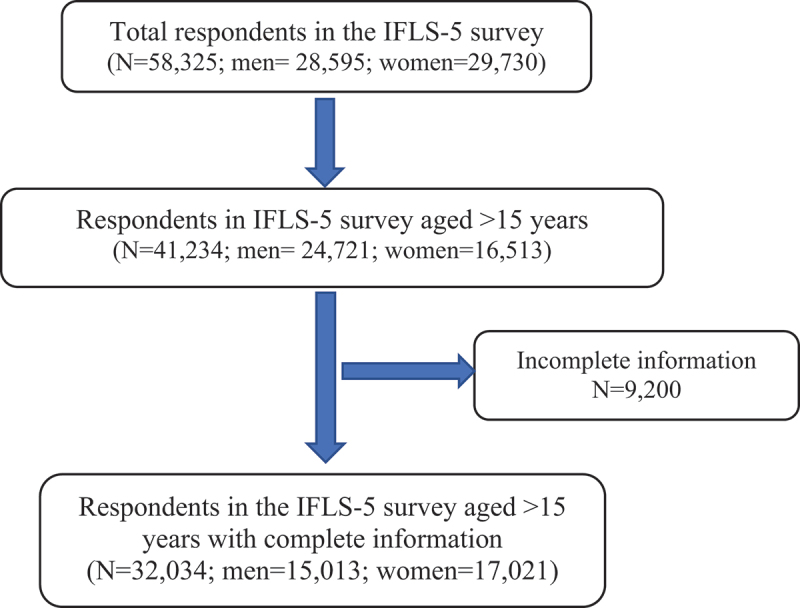
Flowchart presenting the selection of respondents in the study.

We used cross-sectional data from the 2014/2015 Indonesian Family Life Survey (IFLS-5) [15]. IFLS is a longitudinal socioeconomic and health survey, representing approximately 83% of the individuals living in 13 of 33 provinces in Indonesia. The provinces selected were intended to capture the cultural and socioeconomic diversity of Indonesia and maximize the representation of the population. A total of 321 enumeration areas were randomly selected within each of the 13 provinces from a nationally representative sample frame used in the 1993 National Social Economic Survey (Susenas). Details about IFLS are published elsewhere [16]. A total of 41,234 individuals aged 15 years and above were included in the study (22,3% were excluded for missing data). The analysis included 32,034 individuals (Figure 1). Ethics approval was obtained from the RAND Corporation. Informed consent was obtained from all respondents before data collection. Data from the IFLS are publicly available and can be accessed through their website (https://www.rand.org/well-being/social-and-behavioral-policy/data/FLS/IFLS/access.html).

Outcome variables included hypertension prevalence, awareness, treatment, and control. A person was classified as having hypertension if their systolic blood pressure was equal to or above 140 mmHg or their diastolic blood pressure was equal to or above 90 mmHg based on the average of the last two blood pressure readings or if they self-reported use of antihypertensive drugs [16]. Those who reported to have been told by their doctor or another health professional about their hypertension are considered aware of their hypertension. Hypertension treatment was defined as the use of antihypertensive medicines by those aware of their condition. Control of hypertension was defined as systolic blood pressure of 140 mmHg or below and diastolic blood pressure of 90 mmHg or below among those receiving hypertensive treatment.

**Exposures –** Based on questions related to ownership of durable assets and housing infrastructures (sources of drinking water, toilet, garbage disposal, and drainage), socioeconomic status was calculated. Based on these questions, we applied Principal Component Analysis (PCA) [17] to construct a wealth index which is divided into quintiles with the first quintile (Q1) representing the group with the lowest socioeconomic status, and the fifth quintile (Q5) representing the group with the highest status.

Among the socio-demographic variables assessed in the study were age (15–34, 35–54, 55–74, 75+ years), gender (men/women), marriage status (unmarried, married, widowed/divorced), educational level (no formal education, primary and secondary education, and higher education), employment status (employed and unemployed), smoking status (smokers, ex-smokers, non-smokers), health insurance ownership (had/did not have insurance). Body mass index (BMI) was calculated by dividing weight (in kilograms) by the square of height (in square meters) (kg/m^2^). Based on the Asian population cut-off points [18], BMI was categorized as underweight (18.5 kg/m^2^), normal weight (18.5–22.9 kg/m^2^), overweight (23–24.99 kg/m^2^), and obese (>25 kg/m^2^).

### Statistical analyses

Statistical analyses were carried out using Stata 13.0. All analyses were weighed using IFLS survey weights. Weights are calculated by taking into account age, gender, residency, and enumeration area distribution. A descriptive analysis standardized by age was performed to describe the respondents’ characteristics. Univariable and multilevel logistic regression analyses were performed to examine factors associated with prevalence, awareness, treatment, and control of hypertension, adjusted for all covariates (fixed effects with random intercepts). The null model was fitted, resulting in a random effect only model (Supplementary Table S1). The variance partition coefficient (VPC) was calculated to determine the percentage of variance explained by the district level [19]. Model diagnostics were based on likelihood ratio tests. Moreover, we tested for multicollinearity, and the results indicated no multicollinearity.

The concentration index (CI) was used to measure socioeconomic inequality, and the degree and direction of wealth-based inequality were examined in the prevalence, awareness, treatment, and control of hypertension, with ranged from −1 to +1. CI values that are negative imply that prevalence, awareness, treatment, and control of hypertension were more prevalent in lower-income groups. Positive CI values, on the other hand, indicate that outcomes were more concentrated among groups with higher socioeconomic status [20,21]. The sensitivity analysis was conducted using multiple imputed datasets [22] since 22.3% of missing data was observed on some covariates (marital status, education, working insurance income, smoking status, and BMI). Sensitivity analyses showed comparable results with analyses using complete cases (Supplementary Table S1).

## Results

**Figure 2.**
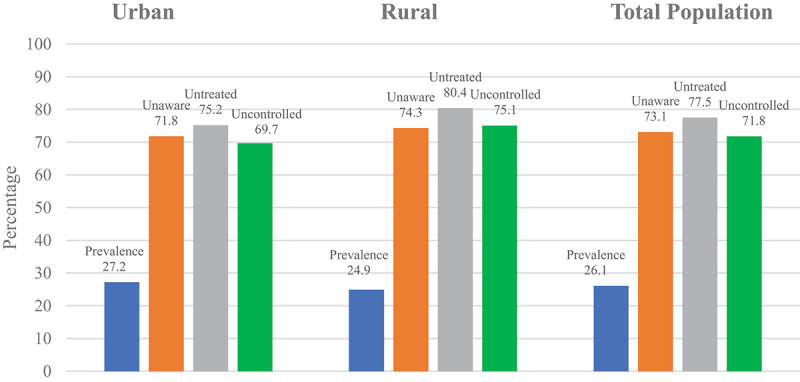
Age-Standardised prevalence, awareness, treatment, and control of hypertension based on residential areas.

In Table 1, the study respondents were more likely females (53.1%), aged 15–34 years old, married, with at least secondary education, lived in urban areas, employed, non-smokers, had BMI less than 23 kg/m^2^ and had health insurance. Only 13.3% had high education, 17.3% in urban and 7.6% in rural areas. Age-standardised prevalence, awareness, treatment, and control of hypertension based in residential areas are shown in Figure 2. Among the 26.1% of people with hypertension, only 26.9% were aware of it, only 22.5% received hypertension treatment, and only 28.2% had their blood pressure controlled. The prevalence of hypertension was similar in rural and urban areas (27.2% vs. 24.9%). There was, however, a greater proportion of unaware, untreated, and uncontrolled hypertension in rural areas than in urban areas (74.3% vs 71.8%; 80.4% vs 75.2%; 75.1% vs 69.7% respectively).

**Table 1.** Characteristics of the IFLS study participants.

Variables	Urban (*N*=18,845)		Rural (*N*=13,189)	
	N	%	N	%
**Gender**				
Male	8,807	46.7	6,206	47.1
Female	10,038	53.3	6,983	52.9
**Age (years)**				
15-34	9,156	48.6	6,154	46.7
35-54	6,865	36.4	4,738	35.9
55-74	2,500	13.3	1,932	14.6
>75	324	1.7	365	2.8
**Marital status**				
Unmarried	4,207	22.3	2,066	15.7
Married	13,044	69.2	9,947	75.4
Widowed/divorced	1,594	8.5	1,176	8.9
**Education level**				
No formal education	548	2.9	1,024	7.7
Primary education	4,475	23.8	5,113	38.8
Secondary education	10,563	56.0	6,054	45.9
Higher education	3,259	17.3	998	7.6
**Employment**				
Unemployed	6,221	33.0	3,792	28.8
Employed	12,624	67.0	9,397	71.2
**Insurance ownership**				
No	8,484	45.0	5,349	40.6
Yes	10,361	55.0	7,840	59.4
**Wealth index**				
1^st^ Quintiles (poorest)	1,964	10.4	3,516	26.7
2^nd^ Quintiles	3,243	17.2	3,059	23.2
3^rd^ Quintiles	4,058	21.5	2,520	19.1
4^th^ Quintiles	4,619	24.5	2,113	16.0
5^th^ Quintiles (richest)	4,961	26.4	1,981	15.0
**BMI (kg/m2**)				
Underweight (<18.5)	2,226	11.8	1,836	13.9
Normal (18.5–22.9)	7,182	38.1	5,926	44.9
Overweight (23–24.9)	2,968	15.8	1,987	15.1
Obese (>25)	6,469	34.3	3,440	26.1
**Smoking status**				
Non-smokers	12,381	65.7	8,106	61.5
Ex-smokers	936	5.0	589	4.5
Current smokers	5,528	29.3	4,494	34.0

Finding from univariate logistic regression are presented in Table 2. Based on the multilevel multivariable logistic regressions (Table 3), a modest amount of variation was observed in the prevalence, awareness, and treatment of hypertension, with VPCs of 2.2%, 2.3%, and 5% respectively. Results from this study show that respondents who were older and male, unmarried, widowed/divorced, without formal and primary education, and unemployed were significantly more likely to have hypertension (p < 0.05). The odds of hypertension were 1.70 (95%CI = 1.56–1.86); 2.94 (95%CI = 2.73–3.17) higher for overweight and obese respondents, respectively. Compared to respondents with the highest socioeconomic status (Q5), respondents with medium socioeconomic status (Q3) had 12% higher odds of hypertension. Smokers had a 15% lower risk for hypertension than non-smokers (OR = 0.84, 95%CI = 0.77–0.92).

**Table 2.** Univariate logistic regression of the factors associated with the prevalence, awareness, treatment, and control of hypertension among the adult population in Indonesia.

Variables	Prevalence (*N*=32,034)	Unaware (*N*=7,623)	Untreated (*N*=2,492)	Uncontrolled (N=676)
	OR (95%CI)	OR (95%CI)	OR (95%CI)	OR (95%CI)
**Age (years)**				
15-34	ref.	ref.	ref.	ref.
35-54	4.29 (3.97–4.63)	0.37 (0.31–0.44)	0.64 (0.42–0.99)	2.33 (1.09–4.99)
55-74	12.1 (11.0–13.3)	0.26 (0.22–0.31)	0.43 (0.28–0.66)	4.66 (2.17–10.0)
>75	28.8 (23.9–34.9)	0.31 (0.24–0.40)	0.53 (0.32–0.88)	8.58 (2.67–27.6)
**Gender**				
Female	ref.	ref.	ref.	ref.
Male	0.88 (0.82–0.93)	2.16 (1.95–2.41)	1.20 (0.98–1.47)	0.80 (0.51–1.24)
**Marital status**				
Married	ref.	ref.	ref.	ref.
Unmarried	0.31 (0.28–0.34)	4.02 (2.99–5.40)	1.83 (0.88–3.81)	0.56 (0.14–2.23)
Widow/Divorced	2.94 (2.70–3.21)	0.75 (0.66–0.84)	0.90 (0.73–1.12)	2.83 (1.60–5.00)
**Residence**				
Urban	ref.	ref.	ref.	ref.
Rural	0.91 (0.86–0.97)	1.20 (1.08–1.33)	1.46 (1.19–1.77)	1.76 (1.08–2.87)
**Education level**				
No formal education	3.79 (3.28–4.38)	0.98 (0.78–1.22)	1.60 (1.08–2.37)	7.71 (2.73–21.7)
Primary education	2.07 (1.87–2.29)	0.96 (0.80–1.15)	1.61 (1.17–2.21)	2.00 (1.08–3.71)
Secondary education	0.88 (0.80–0.97)	1.21 (0.99–1.45)	1.32 (0.96–1.83)	1.10 (0.58–2.07)
Higher education	ref.	ref.	ref.	ref.
**Employment**				
Employed	ref.	ref.	ref.	ref.
Unemployed	1.18 (1.11–1.26)	0.53 (0.47–0.59)	0.62 (0.51–0.76)	1.09 (0.70–1.69)
**Insurance ownership**				
Yes	ref.	ref.	ref.	ref.
No	0.96 (0.91–1.02)	1.35 (1.22–1.50)	1.25 (1.04–1.51)	1.33 (0.85–2.10)
**Wealth index**				
1^st^ Quintiles (poorest)	1.40 (1.27–4.53)	1.56 (1.32–1.85)	2.27 (1.64–3.13)	2.42 (1.09–5.37)
2^nd^ Quintiles	1.10 (1.00–1.21)	1.23 (1.05–1.46)	1.34 (1.01–1.78)	1.44 (0.76–2.74)
3^rd^ Quintiles	1.18 (1.08–1.29)	1.21 (1.03–1.42)	1.29 (0.98–1.70)	0.98 (0.55–1.76)
4^th^ Quintiles	1.04 (0.95–1.13)	1.30 (1.09–1.54)	1.08 (0.82–1.43)	1.15 (0.61–2.17)
5^th^ Quintiles (richest)	ref.	ref.	ref.	ref.
**BMI (kg/m2)**				
Underweight (BMI<18.5)	0.80 (0.71–0.89)	0.98 (0.79–1.23)	1.10 (0.72–1.69)	1.94 (0.68–5.54)
Normoweight (BMI 18.5–22.9)	ref.	ref.	ref.	ref.
Overweight (BMI 23–24.9)	1.56 (1.42–1.70)	0.78 (0.67–0.92)	0.95 (0.69–1.29)	1.08 (0.57–2.04)
Obese (BMI >25)	2.38 (2.23–2.56)	0.65 (0.58–0.74)	0.85 (0.67–1.08)	1.82 (1.10–3.01)
**Smoking status**				
Non-smokers	ref.	ref.	ref.	ref.
Former smokers	1.94 (1.72–2.19)	0.81 (0.67–0.98)	0.75 (0.56–1.01)	0.70 (0.39–1.23)
Current smokers	0.87 (0.81–0.93)	2.42 (2.12–2.75)	1.94 (1.47–2.57)	1.22 (0.61–2.42)

**Table 3.** Multilevel analysis of the factors associated with the prevalence, awareness, treatment, and control of hypertension among the adult population in Indonesia.

Variables	Prevalence (N=32,034)	Unaware (N=7,623)	Untreated (N=2,492)	Uncontrolled (N=676)
	OR (95%CI)	OR (95%CI)	OR (95%CI)	OR (95%CI)
**Fixed effects**				
**Age (years)**				
15-34	ref.	ref.	ref.	ref.
35-54	3.41 (3.14–3.71)	0.47 (0.39–0.57)	0.73 (0.49–1.10)	1.93 (0.88–4.25)
55-74	10.5 (9.45–11.6)	0.29 (0.24–0.36)	0.47 (0.30–0.71)	4.10 (1.78–9.44)
>75	23.1 (18.7–28.4)	0.33 (0.25–0.45)	0.65 (0.37–1.15)	4.34 (1.27–14.8)
**Gender**				
Female	ref.	ref.	ref.	ref.
Male	1.57 (1.43–1.72)	1.66 (1.42–1.94)	1.11 (0.82–1.48)	1.30 (0.65–2.57)
**Marital status**				
Married	ref.	ref.	ref.	ref.
Unmarried	1.20 (1.07–1.34)	2.01 (1.50–2.69)	1.50 (0.72–3.09)	0.60 (0.13–2.82)
Widow/Divorced	1.56 (1.41–1.73)	1.05 (0.91–1.21)	1.02 (0.79–1.31)	1.43 (0.79–2.58)
**Residence**				
Urban	ref.	ref.	ref.	ref.
Rural	0.97 (0.89–1.05)	0.99 (0.88–1.13)	1.16 (0.93–1.45)	1.33 (0.82–2.18)
**Education level**				
No formal education	2.22 (1.88–2.61)	1.26 (0.97–1.64)	1.34 (0.84–2.13)	2.47 (0.82–7.49)
Primary education	1.47 (1.31–1.64)	1.05 (0.86–1.29)	1.45 (1.01–2.06)	1.23 (0.62–2.46)
Secondary education	1.07 (0.96–1.18)	1.02 (0.84–1.23)	1.40 (1.00–1.95)	0.81 (0.43–1.52)
Higher education	ref.	ref.	ref.	ref.
**Employment**				
Employed	ref.	ref.	ref.	ref.
Unemployed	1.22 (1.13–1.31)	0.74 (0.65–0.83)	0.68 (0.55–0.84)	0.90 (0.57–1.43)
**Insurance ownership**				
Yes	ref.	ref.	ref.	ref.
No	0.94 (0.89–1.00)	1.32 (1.18–1.46)	1.13 (0.93–1.38)	1.18 (0.75–1.87)
**Wealth index**				
1^st^ Quintiles (poorest)	1.09 (0.98–1.21)	1.45 (1.21–1.74)	1.85 (1.31–2.60)	1.13 (0.49–2.58)
2^nd^ Quintiles	1.03 (0.93–1.14)	1.21 (1.02–1.44)	1.12 (0.83–1.51)	1.25 (0.67–2.34)
3^rd^ Quintiles	1.12 (1.02–1.23)	1.18 (1.00–1.39)	1.34 (1.01–1.77)	0.79 (0.44–1.42)
4^th^ Quintiles	1.04 (0.95–1.14)	1.28 (1.09–1.51)	1.13 (0.86–1.49)	1.14 (0.63–2.06)
5^th^ Quintiles (richest)	ref.	ref.	ref.	ref.
**BMI (kg/m2**)				
Underweight (BMI<18.5)	0.61 (0.55–0.69)	1.04 (0.84–1.29)	1.06 (0.70–1.60)	1.43 (0.54–3.77)
Normoweight (BMI 18.5–22.9)	ref.	ref.	ref.	ref.
Overweight (BMI 23–24.9)	1.70 (1.56–1.86)	0.90 (0.77–1.06)	0.98 (0.73–1.32)	1.42 (0.77–2.60)
Obese (BMI >25)	2.94 (2.73–3.17)	0.79 (0.69–0.89)	0.89 (0.70–1.13)	2.35 (1.44–3.83)
**Smoking status**				
Non-smokers	ref.	ref.	ref.	ref.
Former smokers	1.08 (0.94–1.24)	0.70 (0.57–0.87)	0.82 (0.57–1.17)	0.46 (0.21–1.01)
Current smokers	0.84 (0.77–0.92)	1.55 (1.31–1.83)	1.47 (1.05–2.08)	1.02 (0.45–2.31)
**Random effects**				
Community (PSU) random variance (SE^a^)	0.075 (0.018)	0.079 (0.023)	0.175 (0.068)	0
Community (PSU) VPC^b^ (%)	2.2	2.3	5.0	0

^a^SE: Standard error, ^b^ VPC: variance partition coefficient.

People aged 35 years and older, unemployed, obese, and ex-smokers were more likely to be aware that they had hypertension. In contrast, male respondents, unmarried people, current smokers, and those without health insurance had a higher likelihood of being unaware of their hypertension. Additionally, smokers and those in the poorest and middle SES groups were less likely to receive treatment for their hypertension (OR = 1.47, 95%CI = 1.05–2.08, OR = 1.85, 95%CI = 1.31–2.60, and OR = 1.34, 95%CI = 1.01–1.77, respectively). Moreover, people aged 55+ years and unemployed who were aware of their hypertension had significantly lower odds of being untreated (53% for those aged 55–74, 35% for those aged 75+ and 32% for those unemployed). Those with uncontrolled hypertension were significantly more likely to be older, have no formal education, and be obese among respondents with hypertension treatments.

### Concentration index of prevalence, awareness, treatment, and control of hypertension

**Figure 3.**
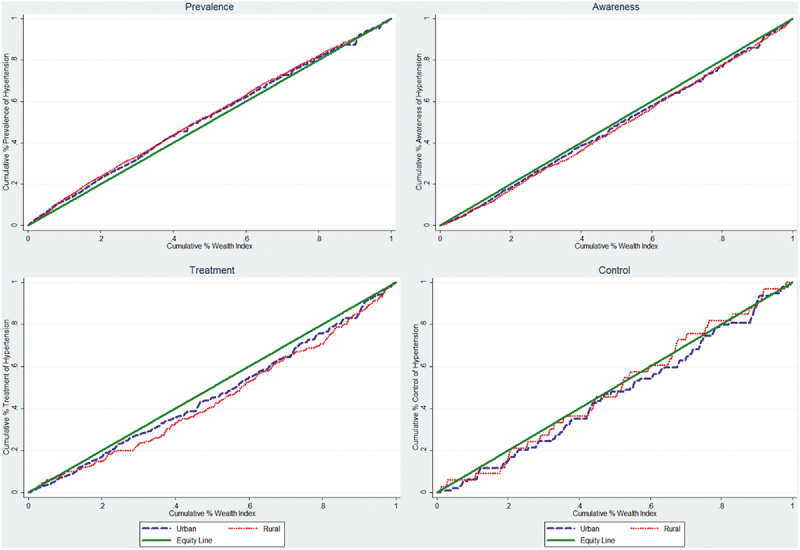
Concentration Curves of prevalence, awareness, treatment, and control of hypertension by wealth index among Indonesian adults in urban and rural areas.

There was a significant negative Concentration Index (CI) for the prevalence of hypertension in total, rural, and urban areas (Table 4), meaning that hypertension was more common among groups with lower socio economic status. The CI values for awareness and treatment of hypertension, on the other hand, showed significant positive trends, indicating that groups with high socioeconomic status were more aware of and treated for hypertension. Despite positive values, the CI for hypertension control was not statistically significant. The concentrate curve (CC), which supports the CI findings, was presented by residential area (urban and rural) in Figure 3 and by total in Supplementary Figure S1. The prevalence of hypertension was more likely concentrated in groups with low socioeconomic status (the concentration curve above the line of equality). Although non-significant, awareness, treatment, and control (which show the concentration curves below the line of equality) showed that the inequality favoured groups with higher socioeconomic status.

**Table 4.** Concentration Index of prevalence, awareness, treatment, and control of hypertension.

	Concentration Index (95% CI)		
	Urban	Rural	Total
Prevalence	−0.047 (−0.069, −0.025)	−0.075 (−0.101, −0.049)	−.0.061 (−0.081, −0.042)
Awareness	0.066 (0.029, 0.103)	0.059 (0.012, 0.107)	0.076 (0.046, 0.107)
Treatment	0.096 (0.030, 0.162)	0.183 (0.087, 0.278)	0.148 (0.094, 0.202)
Control	0.080 (−0.067, 0.227)	0.082 (−0.108, 0.273)	0.090 (−0.021, 0.202)

## Discussion

The current study used the Concentration Index approach to examine the socioeconomic disparities associated with hypertension (prevalence, awareness, treatment, and control) among Indonesian adolescents. To the best of our knowledge, this is the first study that examined the inequality of hypertension across different socioeconomic groups in Indonesia’s adult population. Based on the results of the study, (1) there is a high prevalence of hypertension among the adult population of Indonesia, but low awareness, poor adherence to therapy, and poor control of hypertension among this population, and (2) awareness, treatment, and control of hypertension are more concentrated among individuals with higher socioeconomic status, whereas a high prevalence is among those with lower socioeconomic status. This study fills gaps in the literature that have previously been omitted; socioeconomic inequality is a determinant of public health issue in Indonesia.

The findings of this study show that one in five adults in Indonesia over the age of 15 has hypertension. Less than 50% were aware of the hypertensive, among whom less than 50% were treated. According to these results, awareness, treatment, and hypertension control were significantly lower than the so-called ‘rule of halves’ [23]. Our findings were consistent with those of other studies in Indonesia [4,14]. Increases in risk factors such as tobacco consumption, sedentary lifestyles, and unhealthy diets, including salt intake, may be associated with the rise in hypertension [3,8].

The prevalence of hypertension among Indonesians, particularly in rural areas, was low, as are awareness, treatment, and control of the condition, which is in line with previous studies [4,8]. The geographical difference observed may be due to socioeconomic differences between rural and urban areas. There may be various explanations for the high prevalence of hypertension, including insufficient knowledge of risk factors and prevention methods, lack of availability, access to health care, and the high cost of hypertensive medications. A low awareness of hypertension among the younger generation is concerning, as they have been affected by hypertension at a young age and were not aware of it. Most people with hypertension at a younger age are asymptomatic. It is therefore necessary to target health promotion and intervention activities for the management of hypertension also at a younger age group.

As expected, higher education levels, higher socioeconomic status, older age, BMI >30, and former smokers were more likely to be aware of hypertension in this study. It coincides with the findings from other studies, which showed that older respondents, unemployed respondents, and respondents with higher wealth indices (>Q1) were more likely to receive antihypertensive medication among those who were aware of their hypertension [24–29]. Interestingly, we found that unemployed people are more likely to receive antihypertensive treatment. There is a possibility that the unemployed group included retired civil servants who were covered by the government’s health insurance. However, as of the date of data collection, Jaminan Kesehatan Nasional (national health insurance) has yet to be implemented in Indonesia [30,31]. Further, Indonesian policy has not been enacted effectively to combat the burden of non-communicable diseases (NCDs) [32].

### Socioeconomic inequality of prevalence, awareness, treatment, and control of hypertension

This study demonstrates a socioeconomic disparity in hypertension prevalence, with a higher prevalence among those with lower SES, which is aligned with the previous studies in Iran [33] and a multi-country study [34]. Additionally, Palafox et al. found that hypertension awareness, treatment, and control are more prevalent among those with higher economic status in low-middle- and high-income countries [34]. Some factors other than biological ones may explain this phenomenon, including insufficient healthcare access, chronic occupational and environmental stress, and unhealthy lifestyle choices [35,36]. Moreover, awareness, treatment, and control of hypertension were more prevalent among Indonesian adults from higher socioeconomic strata. Higher socioeconomic status is associated with higher living standards, greater access to information about hypertension care, and better access to medication [37]. The risk of unhealthy behaviours (sedentary physical activity and unhealthy calorie-dense foods) may, however, be greater for these groups.

This study reveals that there was a higher prevalence of hypertension among the rural population and low SES groups. This may be due to the more disadvantaged groups having fewer healthy food choices and consuming more salty and fatty foods [38,39]. Low health literacy and unequal access to health care may contribute to socioeconomic inequalities in health. Poor health literacy leads to lower engagement with health services, a poorer understanding of medications, and worse health outcomes. Programs targeting lower SES groups could potentially reduce health inequalities. In addition, the positive CI values for awareness and control of hypertension were relatively similar in rural and urban populations in Indonesia, suggesting the same pattern of inequality favouring the population with higher SES. Higher SES groups may have better health literacy, which could explain this. As a result, it might increase an individual’s involvement in their own care, which is an important aspect of patient-centred care [40].

### Value and use of the research

The study shows that the burden of hypertension among adult Indonesians is unequally distributed, with a higher burden among the more impoverished population, among whom awareness, treatment, and control of hypertension are suboptimal. Uncontrolled hypertension poses a great risk of complications. Programs for public health promotion of NCDs and hypertension should include this vulnerable and disadvantaged group. The curative, preventive, and promotional aspects of Universal Health Coverage should be implemented for all to ensure equitable access to healthcare services, especially for those in low-income and vulnerable groups [31]. Indonesia is currently implementing a number of national and local initiatives for health promotion programs aimed at reducing the burden of NCDs and hypertension, including Germas (Gerakan masyarakat hidup sehat – community healthy life movement) and Posbindu (Pos pembinaan terpadu – integrated management post).

To improve the outreach of hypertension prevention and control programs, the government should improve health literacy at the individual and community levels as well as access to primary care and family doctors in the community [41,42]. The provision of health education on a healthy diet and lifestyle can also effectively reduce factors related to hypertension [42,43]. The current study and other studies [5,24,44] suggest that education level and socioeconomic status are associated with hypertension, so government programs aimed at reducing or minimizing education level disparities and socioeconomic status in the population may be fruitful [35].

### Strengths and limitations

Since our analysis is based on nationally representative data, the results can be generalized to the Indonesian population, contributing to the existing literature on socioeconomic inequality and hypertension. In addition, the multilevel model used in this study is the most appropriate to test hypotheses about the effect of varying district characteristics on individual hypertension. Using PCA to generate the wealth index of households provides a more reliable estimate of socioeconomic status in Indonesia than income or consumption expenditures. In addition, wealth is more resistant to economic shocks, especially in low- and middle-income countries, due to the high volatility of consumption. In addition, the CI and CC analyses allow us to examine the inequality in the burdens associated with hypertension across the whole socioeconomic distribution rather than just the lowest or highest socioeconomic groups.

There are, however, some limitations to the study. The data used were from a cross-sectional survey. Therefore, associations between determinants and study outcomes must be interpreted carefully. Our study was unable to adjust several factors associated with hypertension diagnosis and treatment, such as salt intake, availability, and access to healthcare facilities for hypertension treatment. Some of the information, such as the history of hypertension and anti-hypertension medications, were self-reported and might be underreported. In light of the economic growth and implementation of the national health insurance plan in Indonesia in recent years, we may assume that hypertension awareness and treatment have improved slightly compared to the current research findings.

## Conclusion

Despite Indonesia’s economic growth, hypertension and its unequal distribution in the population remain significant health problems. Intervention programs for preventing and controlling hypertension that reach a wider population are urgently needed. Therefore, reducing socioeconomic inequality through national health insurance and economic incentive interventions is essential to close the gaps in the prevalence, awareness, treatment, and control of hypertension in Indonesia. The government should also continue the promotive programs for early detection, regular treatment, and control of hypertensive patients, especially those with low socioeconomic status.

## Supplementary Material

Supplemental Material

## Paper context

Hypertension contributes significantly to mortality globally, including in Indonesia. However, few studies investigated the socioeconomic disparity in the prevalence, awareness of treatment, and control of hypertension. This study examines whether the burdens across different socioeconomic groups exist, accounting for variations across geographical areas. Findings reveal that inequality in hypertension exists, indicating effective programs are urgently needed to overcome the problem.
