# Supplementary material for: Socioeconomic disparities in the burden of hypertension among Indonesian adults - a multilevel analysis
Source: Glob Health Action. 2022 Oct 11;15(1):2129131. doi: 10.1080/16549716.2022.2129131 (PMC9559040; doi:10.1080/16549716.2022.2129131)
Supplement: Supplemental Material [file ZGHA_A_2129131_SM6075.docx]

**Appendix**

Appendix to: Socioeconomic disparities in the burden of hypertension among Indonesian adults - a multilevel analysis

**Supplementary Figure 1.** Concentration Curves of prevalence, awareness, treatment, and control of hypertension by wealth index among Indonesian adults for total population


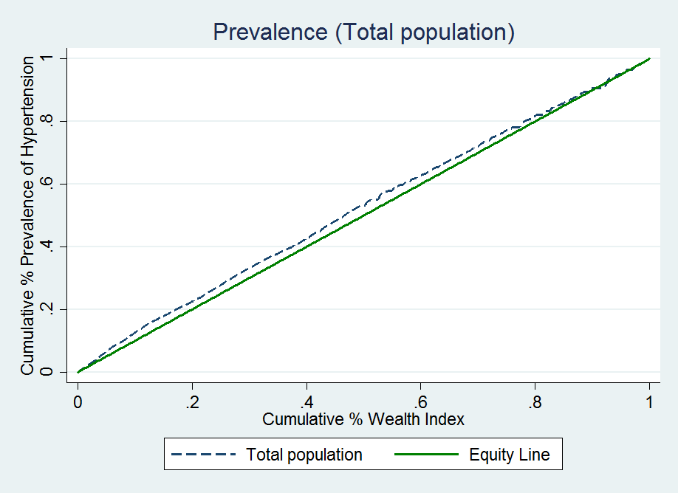

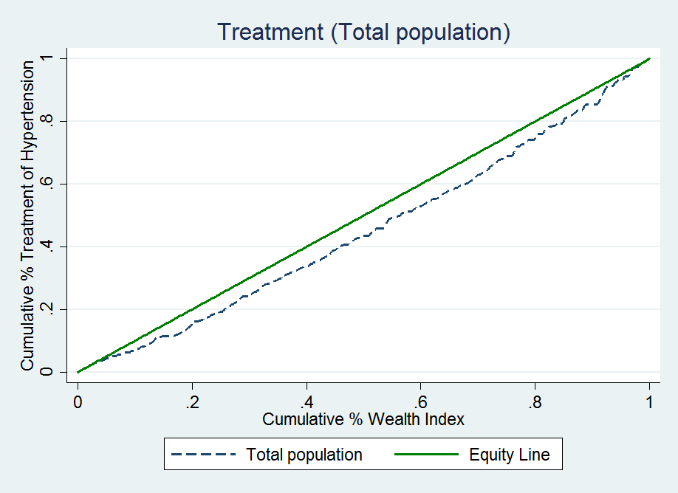

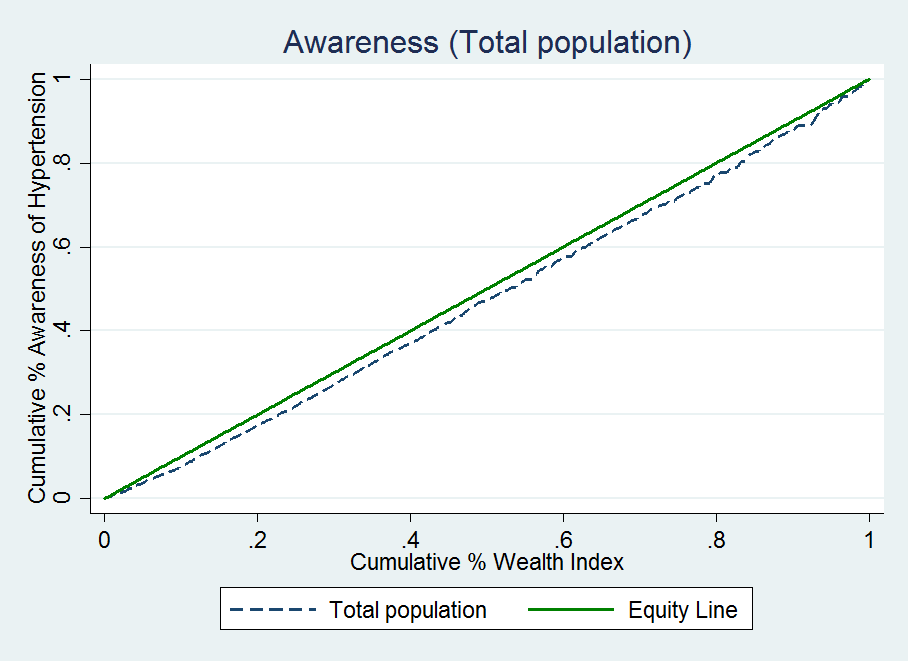

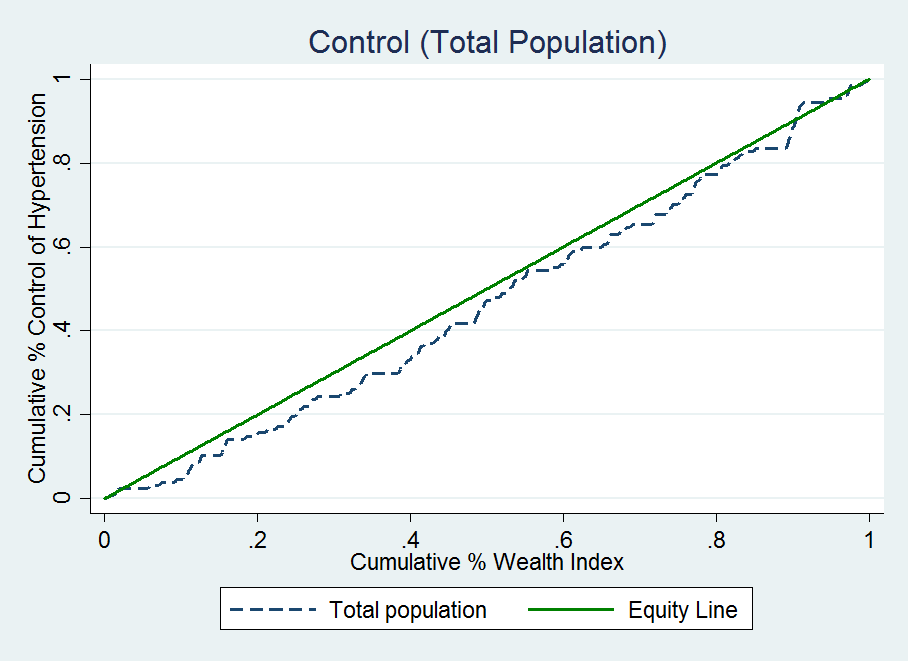


**Supplementary Table 1. Parameter coefficients for the multilevel model for various indicatir of the prevalence, awareenss, treatment and control of hypertension – empty model, without covariates**

|  | Prevalence | Awareness | Treatment | Control |
| --- | --- | --- | --- | --- |
| Random effects | | | | |
| Community (PSU) random variance (SE^a^) | 0.0665 (0.01) | 0.0901 (0.022) | 0.243 (0.074) | 0 |
| Community (PSU) VPC^b^ (%) | 2.0 | 2.7 | 6.9 | 0 |

^a^ SE: Standard error

^b^ VPC: variance partition coefficient

**Supplementary Table 2.** Factors associated with the prevalence, awareness, treatment, and control of hypertension among adult population in Indonesia using multiple imputed data analysis

| **Variables** | **Prevalence** | **Unaware** | **Untreated** | **Uncontrolled** |
| --- | --- | --- | --- | --- |
|  | **OR (95%CI)** | **OR (95%CI)** | **OR (95%CI)** | **OR (95%CI)** |
| **Age (years)**  15-34  35-54  55-74  >75  **Gender**  Female  Male  **Marital status**  Married  Unmarried  Widowed/Divorced  **Residence**  Urban  Rural  **Education level**  Higher education  No formal education  Primary education  Secondary education  **Working status**  Employed  Unemployed  **BMI (kg/m^2^)**  Normal (18.5-22.9)  Underweight (<18.5)  Overweight (23-24.9)  Obese (>25)  **Smoking status**  Non-smokers  Ex-smokers  Smokers  **Insurance ownership**  Yes  No  **Wealth index**  1st quintile (poorest)  2nd quintile  3rd quintile  4th quintile | 1.00  3.34 (3.08-3.62)  10.1 (9.16-11.2)  23.7 (19.7-28.5)  1.00  1.53 (1.40-167)  1.00  1.19 (1.08-1.32)  1.51 (1.37-1.67)  1.00  1.03 (0.96-1.10)  1.00  1.97 (1.66-2.34)  1.46 (1.30-1.63)  1.06 (0.95-1.19)  1.00  1.22 (1.14-1.31)  1.00  0.63 (0.56-0.69)  1.69 (1.53-1.86)  2.89 (2.67-3.13)  1.00  1.08 (0.95-1.23)  0.86 (0.79-.094)  1.00  0.94 (0.88-1.00)  1.11 (1.00-1.23)  1.04 (0.94-1.15)  1.13 (1.03-1.24)  1.03 (0.94-1.12) | 1.00  0.64 (0.58-0.71)  0.40 (0.33-0.49)  0.33 (0.25-0.44)  1.00  1.68 (1.40-2.02)  1.00  2.21 (1.40-3.47)  1.10 (0.92-1.30)  1.00  1.00 (0.94-1.07)  1.00  1.31 (1.04-1.66)  1.04 (0.87-1.24)  1.05 (0.85-1.30)  1.00  0.78 (0.64-0.94)  1.00  1.02 (0.83-1.27)  0.85 (0.69-1.03)  0.76 (0.67-0.86)  1.00  0.67 (0.54-0.84)  1.47 (1.22-1.77)  1.00  1.27 (1.16-1.39)  1.42 (1.14-1.78)  1.16 (0.98-1.38)  1.14 (0.96-1.36)  1.21 (0.98-1.48) | 1.00  0.75 (0.63-0.90)  0.54 (0.38-0.79)  0.47 (0.29-0.74)  1.00  1.14 (0.87-1.50)  1.00  1.49 (0.76-2.94)  1.03 (0.86-1.22)  1.00  0.99 (0.92-1.06)  1.00  1.51 (0.94-2.41)  1.51 (1.20-1.89)  1.39 (0.96-2.00)  1.00  0.69 (0.56-0.86)  1.00  1.19 (0.81-1.77)  0.92 (0.72-1.18)  0.92 (0.68-1.24)  1.00  0.89 (0.67-1.78)  1.45 (1.00-2.12)  1.00  1.18 (0.88-1.59)  1.89 (1.21-2.94)  1.12 (0.89-1.39)  1.24 (0.91-1.67)  1.02 (0.76-1.37) | 1.00  2.10 (1.63-2.70)  4.49 (2.63-7.65)  9.35 (3.90-22.5)  1.00  1.21 (0.59-2.51)  1.00  0.26 (0.05-1.28)  1.14 (0.61-2.14)  1.00  0.99 (0.90-1.07)  1.00  2.84 (1.30-6.20)  1.27 (0.61-2.65)  0.91 (0.44-1.90)  1.00  0.86 (0.65-1.14)  1.00  1.31 (0.62-2.73)  1.73 (1.15-2.58)  2.61 (1.48-4.62)  1.00  0.46 (0.17-1.28)  1.26 (0.45-3.56)  1.00  1.24 (0.83-1.86)  1.71 (0.64-4.60)  1.57 (0.64-3.80)  0.98 (0.38-2.50)  1.24 (0.62-2.51) |
| 5th quintile (richest) | 1.00 | 1.00 | 1.00 | 1.00 |
